# Nature‐Based and Community‐Level Responses to Climate Distress in Young People: A Systematic Review

**DOI:** 10.1002/jad.70171

**Published:** 2026-05-04

**Authors:** Meghana Bhupati, Nita Alexander, Yael Perry, Jack Farrugia

**Affiliations:** ^1^ School of Public and International Affairs Princeton University Princeton New Jersey USA; ^2^ James Cook University Townsville Queensland Australia; ^3^ The Kids Research Institute Australia Nedlands Western Australia Australia; ^4^ The University of Western Australia Nedlands Western Australia Australia; ^5^ Australian Research Centre in Sex Health and Society La Trobe University Bundoora Victoria Australia

**Keywords:** climate distress, community‐level interventions, eco‐anxiety, mental health, nature‐based interventions, youth

## Abstract

**Introduction:**

Climate change is both an environmental crisis and a growing source of psychological distress for young people, calling for responses that nurture emotional resilience and collective engagement. The emerging response to climate distress has mainly focused on formal psychological and individual‐level interventions. However, there is growing recognition that climate distress is shaped by collective, social, and structural conditions, and therefore may also require programs that extend beyond the individual.

**Methods:**

The aim of this systematic review was to examine how nature‐based and community‐level programs support young people experiencing climate distress. A systematic search and synthesis of creative, educational, action‐based, and nature‐focused interventions yielded 11 records, each reporting on distinct interventions. Across studies, participant ages fell within the eligible range of 12–25 years. Programs were conducted in Canada, the United Kingdom, the United States, and globally. Gender was variably reported across studies.

**Results:**

Findings revealed that programs had the most perceived effectiveness when they enabled emotional expression, community connection, and direct engagement with nature. These experiences were reported to transform feelings of isolation and helplessness into belonging, meaning, and agency.

**Conclusions:**

A narrative synthesis of the findings revealed five potential recommendations for future nature‐based and community‐level programs: (i) integrate creative expression, (ii) strengthen community connection, (iii) ground programs in nature, (iv) embed cultural relevance, and (v) build pathways to collective action. Applying these recommendations may enhance the sustainability and impact of future programs and help reduce climate distress among young people through strengthened resilience and shared purpose.

## 1 Introduction

Climate change, defined as the long‐term shift in average temperature and weather patterns driven largely by carbon emissions from human activity, has become both a scientific reality and a major issue of public discourse (United Nations 2024). Global average temperatures have reached approximately 1.5°C above pre‐industrial levels across a 12‐month period, indicating a sustained exceedance of this threshold in recent observations (Copernicus Climate Change Service 2024). This warming is increasing the frequency and severity of extreme weather events such as floods, droughts, and wildfires (Bolan et al. 2024). Beyond environmental impacts, climate change also affects population health, including mental health and wellbeing. Certain groups face disproportionate risks due to socioeconomic, environmental, and demographic factors (Teo et al. 2024; Zhao et al. 2022). Young people, older adults, women, low‐income communities, and Indigenous populations are among the most vulnerable (Durkalec et al. 2015; Zhao et al. 2022).

Adolescents and young people occupy a particularly vulnerable position with respect to the mental health impacts of climate change. They are acutely aware of the crisis that threatens their futures, yet are often excluded from political participation (Alexander et al. 2022). They recognize the importance of individual action while acknowledging the limits of personal influence in addressing systemic challenges (Thomas et al. 2022). Studies show that many report climate distress, including worry, fear, anger, and powerlessness (Jones and Lucas 2023; Mayes and Hartup 2021; Ramadan et al. 2023). Importantly, young people also recognize that attitudes toward climate change are shaped by background and personal experience, meaning that climate distress is experienced differently across social, cultural, and environmental contexts (Wan et al. 2024). These differences are particularly pronounced among Indigenous communities, where environmental change directly affects relationships to land, culture, and identity, with implications for health and wellbeing (Durkalec et al. 2015). For some young people, the scale of the climate crisis feels overwhelming and can lead to helplessness or despair (Burke et al. 2018).

This tension has contributed to the growing phenomenon of climate distress (Seth et al. 2023). Also known as eco‐anxiety or ecological grief, it refers to psychological distress arising from awareness of climate change and its potential impacts (Albrecht et al. 2007; Seth et al. 2023). It may involve fear, sadness, helplessness, or moral conflict (Australian Psychological Society 2019). Climate distress is particularly heightened among young people who perceive their futures as uncertain (Banwell and Eggert 2024). It can emerge without direct exposure to disasters, reflecting the strain of anticipating long‐term environmental change. Persistent distress may interfere with wellbeing (Ballew et al. 2024) and has been linked to anxiety, depression, and diminished agency in youth still developing coping strategies (Padhy et al. 2015; Thomas et al. 2022). Some young people find that engaging in climate‐related activities mitigates these emotions by fostering purpose, agency, and connection with others who share their concerns (Sanson and Bellemo 2021). Climate engagement, whether through activism, education, or community initiatives, can therefore serve as both an emotional outlet and a source of resilience in the face of environmental uncertainty. However, those who feel responsible for protecting the future, such as climate activists, may be especially prone to burnout (Sanson and Bellemo 2021). When their concerns are dismissed or minimized by political or social narratives, feelings of isolation and frustration often intensify (Alexander et al. 2021; Hickman et al. 2021). Over time, sustained environmental anxiety increases vulnerability to mental health difficulties.

Climate‐related mental health impacts can arise through both direct and indirect pathways. Direct impacts refer to psychological effects resulting from lived experiences of climate‐related events, such as extreme weather, displacement, or environmental loss. In contrast, indirect impacts refer to distress arising from awareness of climate change, including exposure to climate‐related information, anticipation of future risks, and concern for the planet and future generations (Berry et al. 2010; Clayton et al. 2017). Emerging literature highlights that climate distress among young people is often driven by these indirect pathways, even in the absence of direct exposure to climate disasters (Hickman et al. 2021). This review focuses specifically on interventions addressing climate distress as a distinct psychological and emotional response to climate change, particularly those targeting indirect or anticipatory forms of distress. This distinction is important, as interventions designed for disaster‐affected populations often address acute trauma and recovery, whereas programs targeting climate distress more broadly tend to focus on meaning‐making, emotional processing, agency, and collective engagement.

In this review, mental health and wellbeing are treated as related but distinct constructs. Mental health refers to psychological symptoms and functioning, including experiences such as anxiety, depression, and climate‐related distress, whereas wellbeing refers more broadly to positive emotional and social functioning, including resilience, coping capacity, connectedness, meaning, and perceived support. This distinction is important because interventions may aim to reduce distress and symptoms, strengthen wellbeing, or address both simultaneously (Dodge et al. 2012; Keyes 2002; World Health Organization 2022).

As the mental health effects of climate change gain attention, there is growing interest in how to support young people experiencing climate distress (Crandon et al. 2024; Daeninck et al. 2023; Salla et al. 2025; Sanson et al. 2019; Williams et al. 2024). Protective factors include psychosocial resources, adaptive coping skills, and opportunities to build agency and connection (Hickman et al. 2021; Vercammen et al. 2023). These factors are also increasingly recognized within the climate adaptation literature, where adaptive capacity is understood not only in material or institutional terms but also as shaped by psychological and social processes, including hope, sense of belonging, and perceived agency. In this context, adaptation can be understood as both a structural and psychosocial process, requiring individuals and communities to develop the emotional and cognitive resources needed to navigate environmental change (Adger et al. 2012; O'Brien and Wolf 2010). Creating and sustaining hope has also been identified as essential (Ojala 2012). Existing reviews primarily examine clinical and psychosocial interventions delivered by trained professionals (Baudon and Jachens 2021; Xue et al. 2024). Clinical interventions include structured therapeutic approaches such as cognitive behavioral therapy, trauma‐informed care, or pharmacological treatment, usually delivered in individual or group formats (Han et al. 2021; Harvey and Gumport 2019). Psychosocial interventions provide structured support to improve emotional wellbeing and coping, including school‐based programs, resilience workshops, or peer‐support groups (Bessell and Moss 2007). While these approaches are valuable, they may not reach those experiencing early or diffuse forms of climate distress. Many young people do not view their distress as a mental health problem or seek help until symptoms significantly affect daily life (Gulliver et al. 2010; Radez et al. 2020). This gap highlights the need for alternative, preventative approaches (Colizzi et al. 2020).

Several intervention studies have focused on individual actions such as reducing energy use or journaling about climate emotions (Haseley 2019; Runkle et al. 2025). However, experts caution that these strategies may place undue responsibility on young people themselves (Hockey 2024). Researchers therefore advocate for holistic, multipronged approaches that include group‐based support and acknowledgment of grief (Baudon and Jachens 2021; Seth et al. 2023). Strong communities and supportive networks that validate young people's emotions are critical for wellbeing (Sanson et al. 2019). Emerging research also points to the benefits of climate‐informed nature engagement, such as reciprocal restoration and social prescribing, for mental health (Baudon and Jachens 2021; Darcy et al. 2025; Thomas et al. 2022).

A growing range of nature‐based and community‐level programs, including outdoor education, restoration efforts, cultural and land‐based healing, and youth‐led activism, have emerged as promising strategies for promoting resilience, connection, and emotional wellbeing (Patrick et al. 2024; Walshe et al. 2023; Wardle 2025). These programs are typically informal and not always delivered by licensed professionals. Nature‐based programs intentionally engage young people in natural environments such as forests, coastlines, and parks to support psychological wellbeing and foster connection with nature (Shanahan et al. 2019). Community‐level programs are grounded in local relationships and cultural contexts, emphasizing shared experience, collective coping, and peer‐based support (Gautam et al. 2024; Haslam et al. 2023; Knez and Eliasson 2017). They offer accessible, low‐cost, and scalable options that can be integrated into schools, local organizations, and youth spaces. Research indicates that nature‐based programs reduce anxiety, enhance restoration, and alleviate depressive symptoms (Chang et al. 2024; Coventry et al. 2021; Nabhan et al. 2020). Community‐level programs that strengthen social connection also build community capacity to prepare for climate impacts (Longman et al. 2023). These approaches provide early, preventative support for young people rather than waiting until distress becomes acute.

Despite their growing relevance, there has been no comprehensive review of the evidence surrounding nature‐based and community‐level programs supporting young people experiencing climate distress. This review systematically examines the current landscape of these interventions, focusing on those that have demonstrated effectiveness. By bringing this evidence together, it aims to identify promising, accessible, and community‐grounded responses that strengthen resilience and mental wellbeing among young people who are concerned about climate change. The remainder of this article outlines the methods used to conduct the review, presents the results of the included studies, and discusses key findings and implications for future research and program development.

## 2 Methods

The systematic review protocol was registered prospectively on June 27, 2025, with PROSPERO, an international database of systematic review protocols for research in health and social care (PROSPERO 2025). Important amendments to the protocol have been documented and made publicly available through the PROSPERO record (CRD420251082059, http://www.crd.york.ac.uk/PROSPERO/). The systematic review was carried out in accordance with the PRISMA 2020 guidelines to ensure transparency and rigor, with the completed PRISMA checklist provided as Supporting Information S1.

### 2.1 Eligibility Criteria

The review included peer‐reviewed empirical studies utilizing any study design, such as randomized control trials, qualitative studies, quasi‐experimental studies, as well as relevant gray literature. Only studies published in English were eligible for inclusion. Eligible studies involved human participants with a mean age between 12 and 25 years. Interventions addressed climate‐related mental health or wellbeing in young people and included a deliberate and substantive nature‐based or community‐level component, either as a primary focus or as a core element of a broader program.

The primary outcomes of interest were improvements in perceived emotional and social support for climate distress, or improvements in a young person's mental health or wellbeing related to climate distress. In this review, mental health referred to psychological symptoms and functioning, including outcomes such as climate‐related distress, anxiety, depressive symptoms, or emotional difficulties, consistent with established definitions (World Health Organization 2022). Wellbeing referred more broadly to positive emotional and social functioning, including resilience, coping capacity, hope, sense of meaning, connectedness, belonging, and perceived support (Keyes 2002; Dodge et al. 2012). Eligible studies therefore reported indicators of youth mental health or wellbeing explicitly linked to experiences of climate distress. Examples included perceived ability to cope with climate‐related emotions, emotional resilience, anxiety or depressive symptoms, social connectedness, or other clearly described wellbeing outcomes. No restrictions were placed on the type of measurement tool used, provided that the outcome was clearly situated in relation to climate distress.

Studies were excluded if they primarily reported on adults over the age of 26 or children under the age of 12. However, participants over 26 years were included if the mean age of participants in the study sample was 25 or under. Studies were excluded if they failed to address climate‐related mental health or wellbeing, examined programs not linked to mental health or wellbeing outcomes, or lacked a measure of effectiveness. Studies limited to clinical or therapeutic interventions in formal healthcare settings, those without nature engagement or community‐based practice, or those addressing crises not explicitly linked to climate change were also excluded. Approaches that focused solely on disaster‐affected young people without explicitly addressing climate‐related distress were not included. While climate‐related disasters can contribute to psychological distress, many studies examining disaster impacts do not conceptualize these experiences within a climate distress framework. Given the distinct and emerging nature of climate distress as a construct, this review prioritized interventions that explicitly engaged with climate‐related emotional responses, including distress arising from awareness, anticipation, or concern about climate change, rather than studies focused exclusively on acute disaster‐related trauma. Studies reporting only curriculum‐based school programs focused exclusively on awareness of or the science of climate change, without addressing coping or emotional responses, were also excluded.

### 2.2 Search Strategy

The search strategy was designed in consultation with a librarian at [University information redacted for anonymity]. An initial search was conducted on Ovid MEDLINE on June 16, 2025, and translated to CINAHL, Embase, PsycINFO, ProQuest, and Web of Science. The concepts of “climate distress,” “young people,” and “mental health” were grouped individually and then combined to retrieve relevant results. The detailed Ovid MEDLINE search string is provided in Appendix A.

The concept of nature‐based or community‐level programs was not included as a specific search term to ensure that informal programs were not missed. Programs that met the inclusion criteria were identified manually during screening. Reference lists of included studies and relevant reviews were hand‐searched to identify additional articles. Citations were imported into Covidence systematic review software for screening, with duplicates removed automatically and verified manually as needed (Covidence 2025).

A gray literature search was conducted to identify programs not documented in peer‐reviewed sources but demonstrating measurable impact. The approach followed Godin et al. (2015), which outlined a systematic methodology emphasizing detailed search planning, multiple complementary strategies, and transparent documentation. A Google search was performed using combinations of “youth/adolescent,” “climate distress,” “climate anxiety,” or “eco‐anxiety,” and “mental health” with filetype:pdf to locate impact reports, program evaluations, and project descriptions published by reputable organizations. The titles and descriptions of the first 100 search results were screened by a single reviewer, and sources meeting inclusion criteria were cited using Zotero and imported into Covidence for full‐text review. Along with Google search results, the review examined the homepages and publication archives of key organizations and agencies. The full list of these organizations is provided in Appendix A. Search terms for gray literature were adjusted as needed to optimize retrieval within Google Scholar, following librarian guidance.

### 2.3 Study Selection and Quality Assessment

Three reviewers were involved in screening academic literature and independently applied the inclusion and exclusion criteria. Initially, two reviewers screened 100 articles, and all three reviewers met to resolve conflicts before proceeding. This ensured a consistent application of criteria before screening progressed. One reviewer then conducted title and abstract screening of all remaining articles using Covidence, with the other two equally sharing the role of second screener. All three reviewers met again to resolve any conflicts. Articles deemed unclear during title and abstract screening automatically proceeded to full‐text review. The full text of the remaining articles was then assessed in a similar process by three reviewers independently to confirm eligibility. Any disagreements were resolved by discussion among all three reviewers, with a majority vote determining the final decision. For gray literature, the two additional reviewers shared responsibility for assessing eligibility, and differences were resolved through discussion until consensus was reached.

The quality of peer‐reviewed studies was assessed using the Critical Appraisal Skills Program (CASP) checklists developed by CASP UK, which guide reviewers through key questions related to study validity, results, and relevance (CASP 2025). CASP provided specific checklists for qualitative research, randomized control trials, and quasi‐experimental studies. Gray literature was assessed using the Authority, Accuracy, Coverage, Objectivity, Date, and Significance (AACODS) checklist (Leena and Ryoko 2017). The assessment process was conducted through Covidence's built‐in quality assessment tool, using a customized template that incorporated both AACODS and the relevant CASP checklists. All eligible studies proceeded to data extraction, and none were excluded on the basis of quality appraisal. Overall, studies were judged to have a low risk of bias, although common methodological limitations were noted, providing reasonable confidence in the reliability of the evidence.

### 2.4 Data Extraction

Data extraction was conducted by a single reviewer and verified by an additional reviewer for accuracy and quality. Data was extracted into a structured table using the Covidence data extraction tool. Extracted information included author details, year of publication, study location, study design, mean age or age range of participants, population type (e.g., high school students, university students, community‐based youth groups), sample size, demographic characteristics (e.g., gender, Indigenous identity, race/ethnicity, socioeconomic status, urban or rural setting), type of program (nature‐based or community‐level, or a combination of both), program description and aims, activities involved, duration and frequency, mode of delivery (e.g., in‐person, hybrid), program facilitators, study outcomes, methods of evaluation, and results related to program effectiveness.

## 3 Results

**Figure 1:**
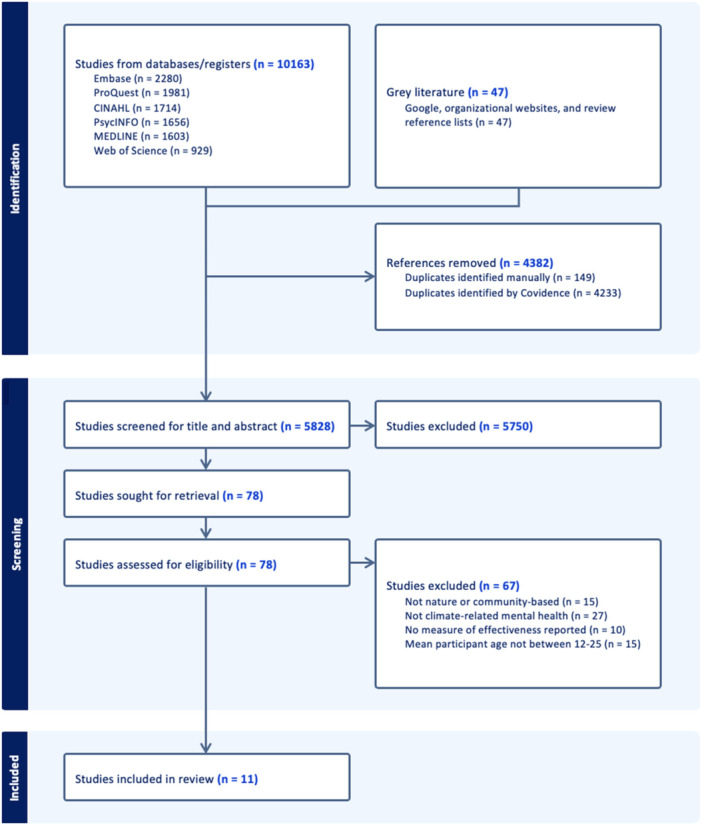
PRISMA diagram adapted from the Covidence platform.

The database search yielded 10,210 references for screening. A total of 4382 duplicates were removed, leaving 5828 studies to be screened by title and abstract. Of these, 5750 were excluded for not meeting eligibility criteria. Seventy‐eight full‐text articles were reviewed in detail, resulting in the exclusion of 67 studies. Eleven studies met the inclusion criteria and were included in this review. The full screening process is outlined in a PRISMA diagram in Figure 1.

### 3.1 Study Characteristics

Most studies (*n* = 10) employed qualitative methodologies, including interviews, focus groups, reflective discussions, and participant reflections (Broadfoot 2024; Cooper et al. 2025; Gallay et al. 2021; Good Grief Network n.d.; Heinemeyer et al. 2024; MacDonald et al. 2015; Marks et al. 2023; Reid and Hanna 2025; Resilience Project 2025; Stewart et al. 2025). One study adopted a mixed methods design that combined validated mental health measures with qualitative data (Epel et al. 2025). Sample sizes ranged from 4 to 486 participants. The mean age of participants across studies fell within the eligible range of 12 to 25 years, with individual study averages ranging from 12.0 years to 23.2 years. Geographically, four studies were conducted in Canada (Cooper et al. 2025; MacDonald et al. 2015; Reid and Hanna 2025; Stewart et al. 2025), three in the United Kingdom (Broadfoot 2024; Heinemeyer et al. 2024; Marks et al. 2023), two in the United States (Epel et al. 2025; Gallay et al. 2021), and two were multinational programs delivered globally (Goof Grief Network n.d.; Resilience Project 2025). Overall, the included studies were published recently, reflecting the emergence of climate distress as a distinct area of research. Most studies were conducted within the past decade, indicating that empirical work examining interventions for climate‐related emotional responses in young people remains limited but is growing. The geographic concentration of studies in high‐income countries, particularly Canada, the United States, and the United Kingdom, further suggests that the evidence base is still developing and may not yet reflect the full diversity of global experiences. Together, these patterns indicate that this is a nascent but rapidly evolving field, with increasing attention to intervention‐based approaches.

### 3.2 Program Types

In this section, the characteristics and reported effectiveness of included programs are discussed by grouping studies into four distinct types. These categories were derived inductively through the analysis, based on the primary features and mechanisms emphasized within each intervention. While many programs incorporated multiple elements, such as education, emotional coping, and action, they were classified according to their dominant feature to facilitate comparison across approaches. This classification is therefore not intended to represent mutually exclusive categories, but rather to highlight the central components through which programs appeared to support young people's mental health and wellbeing in the context of climate distress. Although the studies varied in their aims and delivery, the programs they evaluated consistently sought to support young people's mental health in the context of climate distress. Given that all included studies employed qualitative or mixed methods designs, findings may primarily reflect participants' perceptions of program acceptability, relevance, and perceived impacts, indicating preliminary but promising evidence of potential effectiveness. The four program types identified were: (1) creative programs that used arts‐based methods such as storytelling, theater, or participatory video, (2) school‐ or learning‐based programs that that incorporated emotional reflection and climate‐related themes into educational contexts, (3) action‐based programs that emphasized collective engagement and advocacy, and (4) nature‐focused approaches that involved immersive experiences in outdoor environments to promote restoration, environmental connection, and wellbeing. In this section, programs are described in terms of their defining characteristics and the forms of perceived effectiveness they demonstrated (Table 1).

**Table 1:** Program type definition and examples.

Program type	Definition (Primary feature)	Key characteristics
Creative programs	Programs where artistic and expressive practices are the primary mechanism for processing climate distress and fostering agency	Storytelling, theater, participatory video, creative expression, emotional processing through art, identity, and meaning‐making
School or learning‐based programs	Programs where education combined with emotional reflection is the primary mechanism for addressing climate distress	Structured learning environments, integration of climate knowledge with emotional processing, guided discussion, skill‐building, and classroom or workshop‐based delivery
Action‐based programs	Programs where collective engagement and participation in climate‐related activities are the primary mechanism for transforming distress into agency	Community engagement, advocacy, peer‐led initiatives, environmental restoration, linking emotional processing to action, collective efficacy
Nature‐focused programs	Programs where direct engagement with natural environments is the primary mechanism for supporting wellbeing and reducing climate distress	Outdoor immersion, land‐based learning, ecological connection, restoration of attention, and emotional regulation through nature

#### 3.2.1 Creative Programs

All three creative programs were characterized by their use of artistic expression to help young people process and respond to climate distress (Heinemeyer et al. 2024; MacDonald et al. 2015; Stewart et al. 2025). Evidence from these studies was derived primarily from qualitative methods, including participant interviews, reflections, and facilitator observations. Through storytelling, theater, and participatory video, these interventions created spaces for emotions related to the climate crisis to be expressed, shared, and transformed into agency. In England, the Suitcase Stories project engaged secondary school students in creating and performing narratives about climate adaptation and justice engaged secondary school students in creating and performing narratives about climate adaptation and justice through facilitated, school‐based workshops (Heinemeyer et al. 2024). In Canada, Inuit youth developed participatory videos on climate change through collaborative filming and editing (MacDonald et al. 2015), while another program in Canada engaged Indigenous youth in talking circles and collaborative theater, integrating facilitated group dialog with co‐created performance (Stewart et al. 2025). Heinemeyer et al. (2024) reported that the storytelling process helped participants articulate emotions, reflect on their relationship to climate change, and imagine constructive responses. MacDonald et al. (2015) described how participatory video supported self‐expression, strengthened confidence, and reinforced agency. Stewart et al. (2025) highlighted how Indigenous cultural identity and intergenerational ties deepened belonging, affirmed identity, and strengthened resilience. Across these programs, participants reported improvements in coping, resilience, and agency. Creative expression appeared to help transform emotional responses into solidarity, collective action, and hope, illustrating how art‐based spaces may mobilize both emotional healing and social empowerment.

#### 3.2.2 School or Learning‐Based Programs

All three school or learning‐based programs integrated educational engagement with emotional processing to help students navigate climate distress (Broadfoot 2024; Good Grief Network n.d.; Marks et al. 2023). Findings from these studies were based primarily on qualitative evidence, including student reflections, interviews, and participatory workshop outputs. Learning was positioned not only as acquiring environmental knowledge but also as acknowledging and working through emotions such as sadness, guilt, and fear arising from the climate crisis. In the United States, the Mycelium Youth Network blended Indigenous knowledge with STEAM education through hands‐on, skill‐based projects such as solar construction and air quality monitoring, linking skill‐building to empowerment and resilience (Broadfoot 2024). Delivered online or in person, The Good Grief Network's GGN‐Z Teen Program provided facilitated, multi‐session spaces for adolescents to process and manage complex emotions through guided reflection, journaling, and collective dialog (Good Grief Network n.d.). In the United Kingdom, a co‐designed workshop invited secondary school students to engage in guided discussions and creative scenario‐building, reframing distress into positive visions of sustainable futures (Marks et al. 2023). Across these programs, participants described increased climate understanding, stronger confidence in taking action, and reduced isolation through shared learning. This suggests that educational settings that integrate emotional literacy with collective inquiry thus have the potential to foster both knowledge and wellbeing, transforming classrooms into spaces of collective resilience and active hope.

#### 3.2.3 Action‐Based Programs

All three action‐based programs emphasized collective engagement and advocacy as strategies to transform climate distress into meaningful action (Cooper et al. 2025; Gallay et al. 2021; Resilience Project 2025). Findings from these studies were derived primarily from qualitative evidence, including participant interviews, reflections, and facilitator reports. These initiatives used hands‐on and peer‐support strategies where young people worked together to restore ecosystems, promote sustainability, and influence decision‐making. In Canada, Climate Cafés created peer‐facilitated spaces where youth expressed emotions, shared coping strategies, and engaged in structured group discussion, often linking these reflections to collective engagement and climate‐related action (Cooper et al. 2025). In the United States, civic science projects partnered with schools and community organizations to engage youth in sustained, place‐based activities such as reclaiming abandoned land, monitoring soil and water, and restoring habitats (Gallay et al. 2021). Participants described how visible environmental progress helped alleviate helplessness and transform concern into action. Globally, the Resilience Circles network supported youth climate activists through peer‐led meetings emphasizing emotional resilience, mutual care, and structured reflection to sustain engagement (Resilience Project 2025). Across these programs, participants reported improvements in coping, increased confidence, and sustained climate‐positive behaviors. By combining advocacy with emotional support and community connection, these programs seemed to support young people in reframing distress into agency and maintaining resilience through shared efforts to care for the environment.

#### 3.2.4 Nature‐Focused Programs

Two studies described nature‐focused programs emphasizing direct engagement with natural environments as foundational to emotional resilience (Epel et al. 2025; Reid and Hanna 2025). Evidence from both studies was based primarily on qualitative participant accounts, including reflections and course or program evaluations. Using outdoor immersion and experiential learning, participants processed climate‐related emotions while strengthening their connection to the natural world. In the United States, a university‐level resilience course incorporated outdoor immersion, group projects, reflective practices, and quantitative measures of wellbeing and collective efficacy, enabling students to link wellbeing to shared purpose and environmental responsibility (Epel et al. 2025). In Canada, the Riparia program immersed young women in freshwater expeditions combining ecological science, land‐based knowledge, and hands‐on environmental learning, creating reflective space to explore environmental change and develop coping strategies (Reid and Hanna 2025). Participant accounts across both programs indicated reductions in climate‐related anxiety, improved wellbeing, and a stronger connection to nature and peers. Direct engagement with natural environments, thus, may serve as a protective mechanism against despair, offering emotional restoration and renewed belonging within ecological systems (Table 2).

**Table 2:** Study characteristics and key components of nature‐based and community‐level programs.

Authors (Year), Country	Study design	Methodology	Sample characteristics	Intervention description	Primary aim(s) of program	Key components
Creative programs						
Heinemeyer et al. (2024), England	Qualitative	Qualitative interviews, reflective discussions	25 secondary school students, aged 11–17, all female, mostly first/second‐gen migrant families	Participatory storytelling project (“Suitcase Stories”) exploring climate adaptation and justice	Increase understanding of climate adaptation and empower youth agency	Story creation, intergenerational exchanges, live performances
MacDonald et al. (2015), Canada	Qualitative	Participant reflections, facilitator reports	7 Inuit youth, aged 12–16, 2 male, 5 female	Youth‐led participatory video workshops on climate adaptation	Support adaptive capacity and resilience through creative expression	Filming, editing, video production, peer collaboration
Stewart et al. (2025), Canada	Qualitative	Qualitative interviews	12 Indigenous youth, aged 16–29	Talking circles and a co‐created theater project for Indigenous youth	Create culturally rooted spaces for youth to process climate distress	Talking circles, collaborative playwriting, theatrical performance
School‐ or learning‐based programs						
Broadfoot (2024), USA	Qualitative	Participant reflections	High school students, low‐income, POC	Mycelium Youth Network STEAM‐based climate resilience training for low‐income youth of color	Equip youth with hands‐on skills for climate resilience and agency	DIY air purifiers, solar energy, native planting, STEAM projects
Good Grief Network (n.d.), Worldwide	Qualitative	Participant feedback, reflective journaling	Teenagers, no demographic data available	GGN‐Z Teen Program supporting emotional coping for teens facing climate distress	Provide teens with tools to manage eco‐anxiety and build grounded hope	Journaling, nature meditations, collective visioning, creative arts
Marks et al. (2023), UK	Qualitative	Qualitative interviews	4 secondary school students, aged 16–18	Youth co‐designed storytelling and visioning workshop	Explore eco‐emotions and cultivate realistic, active hope	Storytelling, guided visualization, peer sharing, gratitude exercises
Action‐based programs						
Cooper et al. (2025), Canada	Qualitative	Qualitative interviews	7 university students and young adults, aged 18–24, female, male, non‐binary	Peer‐based Climate Cafés offering facilitated spaces for youth to process climate emotions	Provide safe spaces for emotional expression and build collective resilience	Active listening, group discussion, coping toolkits
Gallay et al. (2021), USA	Qualitative	Focus groups, reflective reports	486 secondary school students, aged 12–18, 59% Black, 17% Latinx, 14% mixed, 76–86% low‐income	Place‐based civic science projects connecting environmental action with community restoration	Foster belonging, agency, and eco‐resilience through collective action	Environmental restoration, park reclamation, stormwater reduction
Resilience Project (2025), Worldwide	Qualitative	Participant feedback, reflective notes	Young adults, aged 18–28, POC and LGBTQ+	Peer‐led Resilience Circles designed for young climate activists	Build collective emotional resilience and prevent burnout	Peer‐led circles, emotional processing, burnout prevention workshops
Nature‐focused programs						
Epel et al.(2025), USA	Mixed methods	Climate distress scales, well‐being scales, and collective efficacy measures	190 undergraduate and graduate students, mean age 23.2 ± 8.5, 71% female, 24% male, 3% non‐binary	Climate Resilience course integrating contemplative practices, climate science, and collective projects	Strengthen emotional resilience and climate engagement in young adults	Contemplative practices, group discussions, nature immersion, collaborative projects
Reid and Hanna (2025), Canada	Qualitative	Post‐program reflections	Diverse young women, aged 13–18	Riparia land‐based freshwater learning program	Inspire environmental stewardship and strengthen the connection with freshwater ecosystems	Outdoor expeditions, river and lake learning, underwater drones, microscopes

## 4 Discussion

The aim of this review was to examine how nature‐based and community‐level programs support young people experiencing climate distress. The studies included in this review varied in their form and focus but were grouped into four categories: (1) creative, (2) school or learning‐based, (3) action‐based, and (4) nature‐focused. Together, these programs demonstrated that connection, community, and agency are important dimensions of supporting young people's wellbeing in the context of climate change.

The findings of this review build on the understanding that nature‐based and community‐level programs are potentially effective because they create opportunities for connection and shared meaning. These findings align with broader climate adaptation literature, which emphasizes that adaptive capacity is not solely determined by infrastructure or policy but also by psychosocial factors such as hope, identity, and collective efficacy. Interventions that foster belonging, meaning, and agency may therefore contribute not only to improved wellbeing but also to the development of a “state of mind” that supports adaptation in the face of environmental change (Adger et al. 2012; O'Brien and Wolf 2010). While climate distress often arises from feelings of isolation and helplessness (Hickman et al. 2021), the programs examined here suggest that collective engagement may transform these emotions into solidarity, agency, and hope. Across program types, effectiveness was reported when interventions enabled participants to express emotions, connect with others, and engage directly with the natural world. It was from these common elements that five potential key recommendations arose (see Table 3) that outline how future programs could apply these insights to strengthen their impact and sustainability. Given that the current evidence base is largely qualitative and descriptive, further research using more robust and comparative study designs is needed to test these recommendations and establish their effectiveness.

**Table 3:** Key recommendations for future programs.

Recommendation	Description
Integrate creative expression	Incorporate arts‐based methods such as storytelling, theater, visual art, or participatory media into program design to support emotional expression and agency.
Foster connection to community	Create peer‐led, intergenerational, and collaborative spaces that encourage dialog, shared purpose, and collective coping.
Ground programs in nature	Include outdoor learning, land‐based activities, or ecological restoration that connect participants directly with natural environments.
Embed cultural relevance	Co‐design programs with local communities, drawing on Indigenous knowledge, place‐based practices, and cultural traditions.
Build pathways to agency and collective action	Link emotional reflection with concrete community or environmental projects that promote empowerment and sustained engagement.

### 4.1 Integrate Creative Expression

Creative expression emerged as a central mechanism through which participants described processing emotions and developing a sense of agency in the face of climate distress. Programs using storytelling, theater, and participatory video seemed to enable young people to articulate feelings of fear and grief, share experiences, and transform distress into engagement (Heinemeyer et al. 2024; MacDonald et al. 2015; Stewart et al. 2025). Such creative approaches are consistent with research showing that artistic and expressive practices support emotion regulation and coping in times of psychological stress (Brooke et al. 2022; Wardle 2025). Within Climate Cafés and Resilience Circles, activities like journaling and collective visioning were noted as reflective tools that sustained wellbeing and fostered empathy (Cooper et al. 2025; Resilience Project 2025). These findings reinforce that creativity may function not only a vehicle for emotional release but a pathway for meaning‐making and shared understanding. By embedding arts‐based elements such as storytelling or performance within educational and community contexts, programs can help young people process emotions collectively and reframe distress as purposeful engagement. Such approaches may also support the cultivation of hope by enabling young people to reframe climate concerns into shared narratives of possibility and future‐oriented thinking.

### 4.2 Foster Connection to Community

Across program types, connection to others was a defining feature of perceived effectiveness. Initiatives that prioritized peer dialog, collaboration, and mutual support were described as helping participants transform isolation into belonging and collective strength. Climate Cafés and Resilience Circles provided structured spaces for shared reflection and collective coping, which participants reported helped normalize climate‐related emotions while fostering solidarity (Cooper et al. 2025; Resilience Project 2025). Similarly, civic science projects and nature expeditions seemed to cultivate intergenerational partnerships and community trust (Gallay et al. 2021; Reid and Hanna 2025). These results align with social identity and resilience theories that emphasize belonging as a key determinant of mental health (Haslam et al. 2023). In the context of climate distress, feeling connected to others enables youth to draw on social support and build confidence in collective efficacy (Ballew et al. 2024). Based on this, future programs could benefit from intentionally creating inclusive, peer‐led environments that value dialog and shared purpose, allowing community connection to serve as both emotional support and a foundation for sustained engagement. These approaches may be particularly effective when embedded within ongoing community initiatives, allowing emotional support to translate into sustained engagement and collective coping over time.

### 4.3 Ground Programs in Nature

Direct engagement with the natural world was a recurring element in participants' accounts of perceived benefit across program types. Across school, community, and outdoor initiatives, spending time in nature appeared to support emotional wellbeing, reduce anxiety, and strengthen environmental connection. The Riparia freshwater expeditions combined ecological science with land‐based learning, fostering understanding and emotional calm through time on the water (Reid and Hanna 2025). Similarly, university resilience courses and civic science projects that involved outdoor immersion helped students translate worry into constructive action (Epel et al. 2025; Gallay et al. 2021). These reported outcomes are supported by broader evidence showing that nature contact enhances mental health and resilience (Coventry et al. 2021; Chang et al. 2024). Direct experiences of nature may counter climate distress by restoring attention, promoting calm, and reinforcing a sense of belonging to the natural world (Shanahan et al. 2019). Future programs may therefore consider including outdoor learning, conservation, or ecological restoration as integral components, grounding emotional support in connection to the environment. Incorporating structured environmental activities, such as restoration or monitoring projects, may further support behavioral engagement while reinforcing connections between wellbeing and environmental stewardship.

### 4.4 Embed Cultural Relevance

Embedding cultural and contextual relevance within program design emerged as an important feature of engagement and long‐term sustainability. Programs co‐designed with local communities, especially Indigenous youth, were described as allowing participants to connect climate experiences to identity, land, and cultural heritage (Stewart et al. 2025). For example, integrating talking circles and cultural storytelling enabled youth to process distress through shared traditions and community knowledge. Similarly, the Mycelium Youth Network's STEAM curriculum connected ecological science with Indigenous ecological practices, enhancing relevance and reinforcing responsibility to place (Broadfoot 2024). These examples align with broader literature showing that culturally grounded approaches enhance participation, belonging, and resilience (Sanson and Bellemo 2021; Thomas et al. 2022). Programs that acknowledge the social, historical, and environmental contexts in which distress occurs may be better positioned to sustain engagement and promote collective stewardship. Future programs that are co‐created with community partners may be likely to ensure alignment with local knowledge, values, and experiences. Programs grounded in local knowledge systems may also strengthen long‐term engagement by aligning climate action with identity, values, and lived experience.

### 4.5 Build Pathways to Agency and Collective Action

Programs were seen to be effective when they supported participants not only to share and process distress but also to transform these emotions into tangible community or environmental projects. Civic science initiatives and Climate Cafés reported that engaging in restoration or awareness activities enhanced young people's sense of control and hope (Cooper et al. 2025; Gallay et al. 2021). Similarly, creative and educational programs linking expression to real‐world outcomes, such as performances or sustainability projects, were described by participants as helpful to move from anxiety to meaningful action (Heinemeyer et al. 2024). These findings are consistent with research demonstrating that agency and collective efficacy protect against climate‐related helplessness and promote wellbeing (Ballew et al. 2024; Salla et al. 2025). When youth perceive that their actions can contribute to change, emotional burden may be reduced and motivation sustained. Future programs may therefore consider integrating emotional reflection with participatory opportunities for collective impact, which can enable young people to transform distress into action that reinforces hope and community resilience. Programs that integrate skill‐building components, such as STEM‐based climate solutions or applied environmental projects, may further support behavioral change while fostering hope through tangible pathways for impact.

## 5 Strengths, Limitations, and Future Directions

This systematic review is the first to examine nature‐based and community‐level programs addressing climate distress among young people. By synthesizing findings across creative, educational, action‐oriented, and nature‐focused programs, it identifies the potential key principles of connection, community, and agency that underpin perceived effectiveness. In doing so, the review moves beyond existing clinical and psychosocial programs to offer practical recommendations for how future programs could be designed and implemented to support young people experiencing climate distress.

These findings should be considered in light of some limitations. The geographic distribution of studies was concentrated in Canada, the United States, and the United Kingdom, with limited representation from the Global South. This restricts the global applicability of the findings and overlooks regional and cultural variations in how community and nature‐based programs may operate. In many Global South contexts, interventions may already integrate local ecological knowledge, intergenerational connection, and collective stewardship in ways not captured in the current evidence base. Future research should therefore examine how such programs function across diverse settings to ensure that mechanisms of connection and resilience are understood globally.

A further limitation concerns the scope of evaluated evidence. This review focused on programs with documented evaluation outcomes, meaning that many promising initiatives were likely excluded. In addition, even when evaluation data were available, outcomes were largely qualitative and descriptive rather than designed to assess formal efficacy or effectiveness. Establishing more rigorous and comparative evaluations should therefore be a priority for future research. As a result, it remains difficult to directly compare effectiveness across different program models.

An additional gap identified in this review is the limited number of interventions explicitly designed for young people experiencing climate‐related disasters. In these contexts, climate distress may be closely intertwined with trauma, loss, and environmental disruption. While disaster‐related mental health impacts may overlap with climate distress, few studies explicitly frame these experiences within a climate distress context. Future research should therefore consider how interventions may be adapted to disaster‐affected settings, including the integration of trauma‐informed approaches, community recovery processes, environmental restoration, and disaster preparedness alongside broader support for climate‐related emotional responses.

Several articles identified in the search described innovative nature‐based or community‐level programs but did not provide formal evaluation data (Grant and Case 2022; Lehtonen and Pihkala 2021; Loose et al. 2023; Nagi et al. 2023). This suggests that a wider ecosystem of effective interventions may exist outside the peer‐reviewed or gray literature. The absence of these evaluations limits understanding of how these programs operate and how their outcomes compare to those reported here. Future research should prioritize the systematic evaluation of such programs, using both qualitative and quantitative methods, and make these results publicly available to inform shared learning. Expanding the evidence base in this way will help refine the mechanisms that make these programs effective and support the development of scalable, inclusive approaches to climate distress. This further reflects the early stage of the field, where intervention‐based research remains limited in scope and geographic diversity.

## 6 Conclusion

This systematic review examined how nature‐based and community‐level programs support young people experiencing climate distress. At the outset, we recognized that climate change affects not only the planet but also the psychological wellbeing of those who will inherit its consequences. By examining programs that connect young people to nature, community, and collective purpose, this review has shown how such programs can transform feelings of isolation and helplessness into belonging, meaning, and action. From this analysis, five potential key recommendations emerged: integrating creative expression, fostering connection to community, grounding programs in nature, embedding cultural relevance, and building pathways to agency and collective action. Together, they suggest that emotional and ecological resilience develop through shared experience, creativity, and care for the natural world. Programs grounded in these principles may improve coping, hope, and engagement among youth facing climate uncertainty. It is essential that more of these initiatives are developed and evaluated, as they represent powerful, evidence‐informed responses to one of the most pressing challenges of our time. Such approaches seem promising to meaningfully reduce climate distress in young people and help build a generation equipped to respond with courage and connection.

## Author Contributions

**Meghana Bhupati:** conceptualization, investigation, writing – original draft, methodology, writing – review and editing, formal analysis, project administration. **Nita Alexander:** conceptualization, investigation, writing – original draft, methodology, writing – review and editing, formal analysis, project administration, supervision. **Yael Perry:** conceptualization, investigation, writing – original draft, writing – review and editing, methodology, formal analysis, project administration, supervision. **Jack Farrugia:** conceptualization, investigation, writing – original draft; methodology, writing – review and editing, formal analysis, project administration, supervision.

## Funding

The authors have nothing to report.

## Ethics Statement

Ethics approval was not required for this study as it involved a review of existing empirical literature and did not include the collection of primary data.

## Conflicts of Interest

The authors declare no conflicts of interest.

## Supporting information

Supporting File:

## Data Availability

Data sharing is not applicable to this article as no datasets were generated or analyzed during the current study.

## Appendix A

Full Search Strategy for Academic Literature

The final search was conducted on 16 June 2025. The search combined the core concepts of *climate distress*, *young people*, and *mental health*.

**Ovid MEDLINE**

((climate adj1 (anxiet* OR distress OR despair OR burnout OR emotion*))

OR (eco‐anxiety OR ecological grief OR environmental anxiety)

OR ((ecological OR environmental) adj3 doom)

OR disaster*

OR ((climate adj1 change*) OR “global warming” OR climate crisis))

AND ((teen* OR youth* OR adolescen* OR (young adj (adult* OR person* OR people*)))

OR ((child* OR kid*)))

AND (((emotional OR mental OR psychological OR psychosocial) adj2 (distress OR health OR wellbeing OR “well‐being” OR wellness OR “well‐ness”))

OR ((resilience OR wellbeing OR wellbeing OR “post traumatic stress” OR “post‐traumatic stress” OR “post traumatic growth” OR “post‐traumatic growth”))).

Search terms were adapted for each database to reflect controlled vocabulary differences. Reference lists of all included studies and relevant reviews were also hand‐searched to identify additional articles. All search results were imported into Covidence systematic review software for screening and de‐duplication (Covidence 2025).

**CINAHL**

S4 AND S7 AND S10

S10, S8 OR S9, 474,570

S9, XB (((emotional OR mental OR psychological OR psychosocial) N2 (distress OR health OR wellbeing OR “well‐being” OR wellness OR “well‐ness”))) OR XB ((resilience OR wellbeing OR well‐being OR “post traumatic stress” OR “post‐traumatic stress” OR “post traumatic growth” OR “post‐traumatic growth”)) OR XB ((“psychosocial recovery” OR (“social” N3 “emotional” N1 “well‐being”) OR (“social” N3 “emotional” N1 “wellbeing”))), 439,402

S8, MH mental health OR MH psychological well‐being, 113,452

S7, S5 OR S6, 2,348,751

S6, TX ((teen* OR youth* OR adolescen* OR (young N2 (adult* OR person* OR people*)))) OR TX ((child* OR kid*)), 2,348,751

S5, MH adolescence OR MH young adult, 774,784

S4, S1 OR S2 OR S3, 26,032

S3, (XB (climate N1 (anxiet* OR distress OR despair OR burnout OR emotion*)) OR XB (eco‐anxiety OR ecological grief OR environmental anxiety) OR XB ((ecological OR environmental) N3 doom) OR XB disaster* OR XB ((climate N1 change*) OR “global warming” OR climate crisis)) AND (S1 OR S2), 24,099

S2, XB ((climate N1 (anxiet* OR distress OR despair OR burnout OR emotion*))) OR XB ((eco‐anxiety OR ecological grief OR environmental anxiety)) OR XB (((ecological OR environmental) N3 doom)) OR XB disaster* OR XB (((climate N1 change*) OR “global warming” OR climate crisis)), 24,099

S1, MH climate anxiety OR MH climate change, 4,811

**ProQuest**

S5, [S1] AND [S2] AND [S3]

Select item 3, S3, (MAINSUBJECT.EXACT(“Mental health”) OR MAINSUBJECT.EXACT(“Child & adolescent mental health”)) OR noft((“mental health” OR “psychological resilience” OR ((emotional OR mental OR psychological OR psychosocial) NEAR/2 (distress OR health OR wellbeing OR “well‐being” OR wellness OR “well‐ness”)) OR (resilience OR wellbeing OR “well‐being” OR “post traumatic stress” OR “post‐traumatic stress” OR “post traumatic growth” OR “post‐traumatic growth”) OR (“psychosocial recovery” OR (“social” NEAR/3 “emotional” NEAR/1 “wellbeing”) OR (“social” NEAR/3 “emotional” NEAR/1 “well‐being”)))), 17 databases, 4,661,261

Select item 2, S2, (MAINSUBJECT.EXACT(“Young adults”) OR MAINSUBJECT.EXACT(“Teenagers”) OR MAINSUBJECT.EXACT(“Children & youth”)) OR noft((teen* OR youth* OR adolescen* OR (young NEAR/2 (adult* OR person* OR people*)) OR child* OR kid*)), 17 databases, 42,776,988

Select item 1, S1, MAINSUBJECT.EXACT(“Climate change”) OR noft((climate NEAR/1 (anxiet* OR distress OR despair OR burnout OR emotion*))) OR noft((“eco‐anxiety” OR “ecological grief” OR “environmental anxiety”)) OR noft(((ecological OR environmental) NEAR/3 doom)) OR noft(disasters*) OR noft((“climate change*” OR “global warming” OR “climate crisis”)), 17 databases, 4,170,220

**Web of Science**

(“mental health” OR “psychological resilience” OR ((emotional OR mental OR psychological OR psychosocial) NEAR/2 (distress OR health OR wellbeing OR “well‐being” OR wellness OR “well‐ness”)) OR (resilience OR wellbeing OR “well‐being” OR “post traumatic stress” OR “post‐traumatic stress” OR “post traumatic growth” OR “post‐traumatic growth”) OR (“psychosocial recovery” OR (“social” NEAR/3 “emotional” NEAR/1 “wellbeing”) OR (“social” NEAR/3 “emotional” NEAR/1 “well‐being”)))

AND

(teen* OR youth* OR adolescen* OR (young NEAR/2 (adult* OR person* OR people*)) OR child* OR kid*)

AND

(“climate change*” OR “global warming” OR “climate crisis” OR disasters* OR (climate NEAR/1 (anxiet* OR distress OR despair OR burnout OR emotion*)) OR “eco‐anxiety” OR “ecological grief” “environmental anxiety” OR ((ecological OR environmental) NEAR/3 doom))

## Appendix B

Search Strategy for Gray Literature

A gray literature search was conducted following the systematic approach outlined by Godin et al. (2015). A targeted Google search was performed using the following terms:

youth OR adolescent (“climate distress” OR “climate anxiety” OR “eco anxiety”) “mental health” filetype:pdf

The search focused on impact reports, evaluations, and project descriptions published by reputable organizations. The first 100 results were screened by title and description, and eligible documents were imported into Covidence for full‐text review.

In addition to Google, the review examined the homepages and publication archives of key organizations and agencies, including:

- Children & Nature Network
- Global Youth Biodiversity Network
- Australian Youth Climate Coalition
- School Strike 4 Climate Australia
- United Nations Environment Program
- 350.org
- Seed Indigenous Youth Climate Network
- Planet Youth
- Young & Resilient Research Center
- Nature and Health Alliance
- Climate Mental Health Network
- Youth and Environment Europe
- Arab Youth Development Network
- Ghana Youth Environment Movement
- Indigenous Youth Roots
- Liter of Light
- Climate Fresk
- Jane Goodall Institute
